# Early embryonic development of Johnston’s organ in the antenna of the desert locust *Schistocerca gregaria*

**DOI:** 10.1007/s00427-022-00695-2

**Published:** 2022-09-23

**Authors:** George Boyan, Erica Ehrhardt

**Affiliations:** 1 Graduate School of Systemic Neuroscience, Biocenter, Ludwig-Maximilians-Universität München, Grosshadernerstrasse 2, 82152 Munich, Planegg-Martinsried Germany; 2 Institute of Zoology, Universität Zu Köln, Zülpicher Str. 47b, 50674 Cologne, Germany

**Keywords:** Locust, Embryo, Development, Antenna, Johnston’s organ

## Abstract

Johnston’s organ has been shown to act as an antennal auditory organ across a spectrum of insect species. In the hemimetabolous desert locust *Schistocerca gregaria*, Johnston’s organ must be functional on hatching and so develops in the pedicellar segment of the antenna during embryogenesis. Here, we employ the epithelial cell marker Lachesin to identify the pedicellar domain of the early embryonic antenna and then triple-label against Lachesin, the mitosis marker phosphohistone-3, and neuron-specific horseradish peroxidase to reveal the sense-organ precursors for Johnston’s organ and their lineages. Beginning with a single progenitor at approximately a third of embryogenesis, additional precursors subsequently appear in both the ventral and dorsal pedicellar domains, each generating a lineage or clone. Lineage locations are remarkably conserved across preparations and ages, consistent with the epithelium possessing an underlying topographic coordinate system that determines the cellular organization of Johnston’s organ. By mid-embryogenesis, twelve lineages are arranged circumferentially in the pedicel as in the adult structure. Each sense-organ precursor is associated with a smaller mitotically active cell from which the neuronal complement of each clone may derive. Neuron numbers within a clone increase in discrete steps with age and are invariant between clones and across preparations of a given age. At mid-embryogenesis, each clone comprises five cells consolidated into a tightly bound cartridge. A long scolopale extends apically from each cartridge to an insertion point in the epithelium, and bundled axons project basally toward the brain. Comparative data suggest mechanisms that might also regulate the developmental program of Johnston’s organ in the locust.

**Supplementary Information:**

The online version contains supplementary material available at 10.1007/s00427-022-00695-2.

## Introduction

Johnston’s organ (JO), first described as an auditory organ of the antenna of a mosquito (Johnston 1855), has since been shown to detect air displacement and so function as an antennal auditory organ in insect species as diverse as *Drosophila* (Göpfert and Robert 2001a, 2002; Göpfert et al. 2002; Todi et al. 2004; Eberl and Boekhoff-Falk 2007; Jarman 2014), cockroaches (Toh 1981; Toh and Yokohari 1985), mosquitos (Göpfert and Robert 2001b), bugs (Jeram and Pabst 1996), ants (Grob et al. 2021), and not least, the desert locust *Schistocerca gregaria* (Gewecke 1972, 1979; Chapman 1982). Anatomical studies confirm a basic ground plan for the JO that is conserved but where the complement of cell clusters may still vary between species (see Lai and Orgogozo 2004; Jarman 2014; Grob et al. 2021).

From a developmental perspective, the JO in *Drosophila* is constructed over the larval to pupal stages during metamorphosis (see Boekhoff-Falk 2005; Eberl and Boekhoff-Falk 2007; Jarman 2014) but only functions effectively as an auditory organ in the adult (see Göpfert and Robert 2001a, 2002). In orthopteroid insects with a hemimetabolous lifestyle, on the other hand, mechanosensory structures such as the JO must be functional on hatching and so need to develop during embryogenesis. Given the significance of this structure for the behavioral repertoire of the locust (see Gewecke 1972, 1979), the absence of any modern developmental literature of which we are aware is surprising.

In this initial study, therefore, we employ immunolabeling coupled with anatomical reconstructions based on confocal microscopy to investigate the early cellular development of the JO in the locust *Schistocerca gregaria*. We establish its epithelial domain of origin, identify putative sense-organ precursors, and analyze the growth of the neuronal lineages these generate. We show that the organization of cell clusters comprising the JO at mid-embryogenesis already reflects that in the adult. We then compare the pattern of development here with that reported for the JO in some other insects.

## Materials and methods

### Animals and preparation

Eggs were obtained from a crowded colony of *Schistocerca gregaria* maintained as previously described (Ehrhardt et al. 2015a, b, 2016), and embryos staged to the nearest 1% of the developmental time (5% = 24 h) according to Bentley et al. (1979).

Protocols for immunolabeling with primary and secondary antibodies, the composition of incubation media, incubation conditions, confocal, fluorescence and Nomarksi microscopy, and image processing were all as previously described (see Boyan and Williams 2004; Ehrhardt et al. 2015a, b, 2016).

### Primary antibodies

*Anti-horseradish peroxidase* (α-HRP, polyclonal rabbit, and Dianova) recognizes a neuron-specific epitope in insects (see Jan and Jan 1982). *Anti-Lachesin* (Mab 1C10, mouse, and gift of M. Bastiani) recognizes a GPI-linked cell surface molecule belonging to the Ig superfamily (see Karlstrom et al. 1993). The expression occurs initially on all differentiating epithelial cells, but only cells involved in neurogenesis, such as precursors continue to express the molecule later. *Anti-Lazarillo* (Mab 10E6, mouse, and gift of D. Sánchez) recognizes a glycosylphosphatidylinositol (GPI)-linked cell surface lipocalin expressed by sensory and pioneer neurons in the grasshopper embryo (Sánchez et al. 1995; Ganfornina et al. 1995). *Anti-phospho-histone H3* (Ser10*,* rabbit, and Millipore) binds the phosphorylated form of the amine terminal of Histone 3 so that staining is only possible when the chromatin lies dissociated from the nucleosome complex, as occurs during mitotic chromosome condensation, and is strongest in the metaphase of the cell cycle (see Hendzel et al. 1997).

### Secondary antibodies

Single staining involved: Alexa® 488 (goat anti-rabbit, Invitrogen) or Cy3 (goat anti-rabbit, Dianova) for fluorescence-based α-HRP; peroxidase (PO)-conjugated goat anti-rabbit (Jackson ImmunoResearch) for Nomarski-based α-HRP followed by counterstaining with diaminobenzidine (DAB) using Sigma Fast DAB tablets; Cy3 (goat anti-mouse, Dianova) for α-Lachesin and α-Lazarillo; Cy3 (goat anti-rabbit, Dianova) for α-PH3. Double-staining (α-HRP/α-Lazarillo) involved Alexa® 488 (goat anti-rabbit, Invitrogen) for α-HRP and Cy3 (goat anti-mouse, Dianova) for α-Lazarillo. Triple-labeling (α-HRP/α-Lach/α-PH3) involved Cy5 (donkey anti-goat, Dianova) for α-HRP, Alexa® 488 (donkey anti-mouse, Invitrogen) for α-Lach, and Cy3 (donkey anti-rabbit, Dianova) for α-PH3.

Controls for the specificity of all secondary antibodies involved (a) the lack of a staining pattern in the absence of the primary antibody and (b) in all cases, a staining pattern consistent with previously published data (see above).

## Results

### Sensory apparatus of the antennal base

**Fig. 1:**
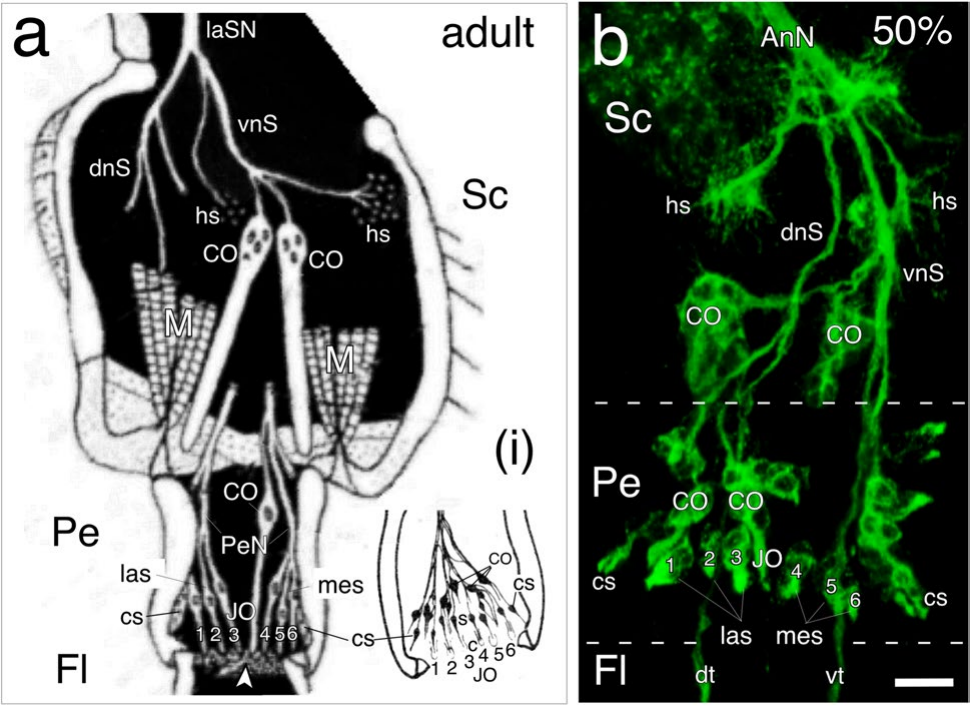
Epithelial domains and the sensory apparatus at the antennal base. **a** Schematic (not to scale, modified with permission from Gewecke 1972) showing principal sensory nerves (laSN, lateral scapal nerve; dnS, dorsal nerve of the scape; vnS, ventral nerve of the scape), sense organs (hs, sensory hair sensilla; CO, chordotonal organs; cs, campaniform sensilla), and muscles (M) in the basal segments (Sc, scape; Pe, pedicel; Fl, flagellum) of the adult antenna. Johnston’s organ (JO) in the Pe comprises symmetrical dorsal and ventral sets of cell clusters (only dorsal shown here), each set containing 6–7 scolopales subdivided into medial (meS) and lateral (laS) fields. Scolopales insert into a membrane (white arrowhead) at the pedicellar/flagellar border, and their axons project basally to respective dorsal or ventral pedicellar nerves (PeN). Inset (**i**): drawing (modified with permission from Gewecke 1979) shows chordotonal sensilla (CO), campaniform sensilla (cs), and six scolopales (s) with cap cells (c) from the dorsal cluster of Johnston’s organ (JO) in the adult pedicel. **b** Confocal image following neuron-specific α-HRP labeling shows the sensory apparatus in the scape and pedicel of the antenna as viewed from the ventral at 50% of embryogenesis. Note the overall similarity to the adult (c.f. Figure 1a) with respect to both innervation patterns, distribution of sensory organs, and the clusters of sensilla of the JO. Parallel ventral (vt) and dorsal (dt) tracts of sensory fibers from the sensilla of the flagellum (Fl) project to the deutocerebrum via the antennal nerve (AnN). The scale bar in b represents 35 µm

The locust antenna is a true appendage (c.f. leg: Gibson and Gehring 1988; Casares and Mann 1998), comprising three articulations: a basal scape that links the antenna to the head capsule, a short intermediary pedicel, and a distal elongated flagellum which is not actively motile (Fig. 1a; see Gewecke 1972, 1979; Chapman 1982). The antennal flagellum is also subdivided into articulations, but these do not represent true segments and so have been termed meristal annuli (Chapman 2002). The major nerve root of the antenna is the antennal nerve which conducts sensory fibers originating from mechanosensory hairs, proprioceptive chordotonal organs, campaniform sensilla, and olfactory sensilla from all three segments to the deutocerebrum of the brain (for a full description see Chapman and Greenwood 1986; Ochieng et al. 1998; Chapman 2002). In addition, the scape possesses a musculature to move the pedicel (Fig. 1a), and this musculature is innervated by axons from motoneurons in the brain (see Gewecke 1972, 1979).

The JO is located in the pedicellar segment and in the adult locust contains symmetrical ventral and dorsal cell clusters, each of which are then subdivided into medial and lateral fields. An adult cluster comprises six to seven scolopales, which insert into a membrane at the pedicellar/flagellar junction (Fig. 1a; inset, i). The cell clusters project axons into pedicellar nerves that run basally to fasciculate with respective medial or lateral nerves in the scape and then join the antennal nerve (Fig. 1a; see Gewecke 1972, 1979 for details). In the hemimetabolous locust, the JO must be functional on hatching and so develops during embryogenesis. Labeling with neuron-specific α-HRP reveals that the innervation pattern, distribution of sensory organs, and organization of clusters of sensilla in the JO existing in the antennal base at mid-embryogenesis (Fig. 1b) already bears a great similarity to that of the adult (c.f. Figure 1a). Our findings suggest that major developmental steps building the JO occur prior to mid-embryogenesis. We, therefore, decided to limit the scope of our present study, which involved screening a total of 235 preparations, to this same time span.

### Epithelial domains

**Fig. 2:**
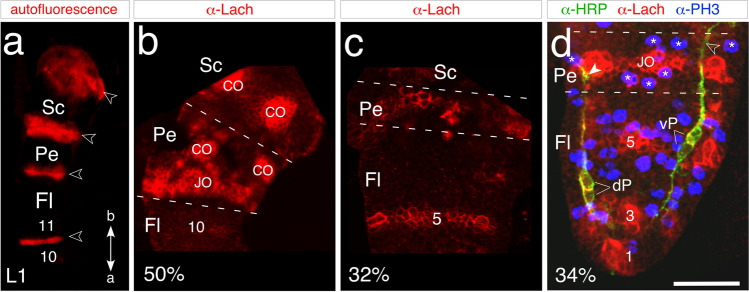
Identifying the pedicellar epithelial domain of the antenna. **a** Photomicrograph of the antennal base in wholemount at the first larval stage (L1) viewed under epifluorescence illumination (false color). Autofluorescence septal-like cuticular bands (open white arrowheads) delimit the scape (Sc), pedicel (Pe), and flagellum (Fl). The most basal meristal annuli (10, 11) at this age have differentiated. Coordinates point to the apex (a) and base (b) of the antenna and apply throughout. Panel modified from Boyan and Ehrhardt 2020. **b** Confocal image of the antennal base at 50% of embryogenesis following labeling against Lachesin (α-Lach, red) reveals discrete Lach-positive epithelial domains delimiting the Sc, Pe, and Fl (10 annuli have differentiated to this point). The segmental bands of Lach-expression already match the cuticular articulation seen at hatching (c.f. panel a). Note the clusters of Lach-positive epithelial cells from which chordotonal organs (CO) and Johnston’s organ (JO) develops. **c** Confocal image of the antennal base at 32% of embryogenesis following labeling against Lachesin (α-Lach, red) reveals the stripe of epithelium representing the early pedicellar domain (segmental borders are dashed white). A further domain (annulus 5) of the Fl has also differentiated. **d** Confocal image of the antenna at 34% of embryogenesis following triple immunolabeling against epithelial cell-specific Lachesin (α-Lach, red), the mitosis marker phosphohistone-3 (α-PH3, blue), and neuron-specific horseradish peroxidase (α-HRP, green). Mitotically active precursors are clearly associated with the serial epithelial domains in annuli 1, 3, and 5 of the flagellum (Fl) and in the pedicel (Pe, white stars) where Lach-positive cell clusters of the future JO are visible. Paired dorsal (dP) and ventral (vP) pioneer neurons have differentiated in the apical flagellum (Fl) and direct axons (white arrowhead, open white arrowhead respectively) through the Pe toward the antennal base. Note that no neurons have differentiated in the pedicel itself at this stage. Panel modified from Boyan and Ehrhardt (2020). The scale bar in d represents 330 µm in a; 120 µm in b; 70 µm in c; 55 µm in d

The pedicel, along with the other articulations of the antenna, can be identified at the end of embryogenesis via epifluorescence illumination, which causes the septal-like cuticular bands to autofluorescence (Fig. 2a). However, this method only functions with cuticularization (after mid-embryogenesis) and does not reveal early epithelial domains. Immunolabeling against the GPI-linked cell surface antigen Lachesin, however, indicates the epithelial domain of the pedicel at all embryonic stages. At mid-embryogenesis (Fig. 2b), Lachesin expression in the scape and pedicel clearly match the segmentation of the antennal base revealed postembryonically by epifluorescence illumination (c.f. Figure 2a) and so allows their unambiguous identification. Still earlier in embryogenesis (32%, Fig. 2c), prior to the formation of the JO, a stripe of Lach-positive cells marks the epithelial domain of the pedicel, while a further stripe representing annulus 5 is visible in the flagellum. Triple immunolabeling against epithelial cell-specific Lachesin (α-Lach, red), the mitosis marker phosphohistone-3 (α-PH3, blue), and neuron-specific horseradish peroxidase (α-HRP, green) at a slightly later stage (34%, Fig. 2d) firstly allows mitotically active precursors associated with the epithelial domains in the flagellum and pedicel to be visualized. The Lach-positive cell clusters of the future JO are also visible in the pedicel, and while HRP-positive dorsal (dP) and ventral (vP) pioneer neurons have differentiated in the apical flagellum, we find no differentiated neurons in the pedicel itself at this stage. We subsequently employed this triple labeling protocol to document the cellular development of the JO from its earliest origins within the molecularly identifiable pedicellar region of the antenna. Our analysis here initially focuses on the ventral epithelium but applies equally to the dorsal region (see Fig. 6 below).

### Sense organ precursors and initial lineages of Johnston’s organ

**Fig. 3:**
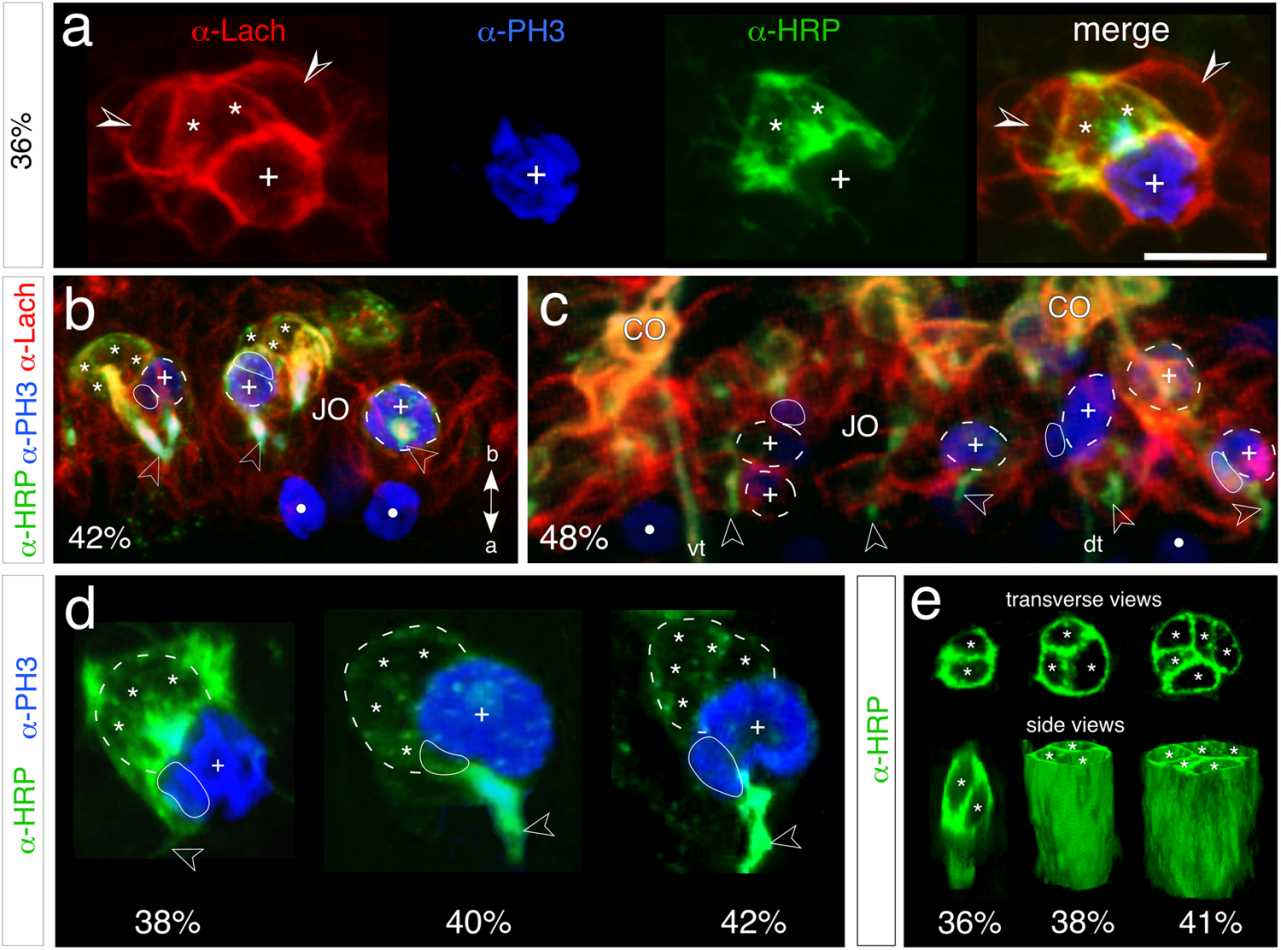
Sense-organ precursors (SOPs) and neuronal lineages of Johnston’s organ (JO) in the pedicellar domain. Coordinates in panel b point to the apex (a) and base (b) of the antenna and apply throughout. **a** High-resolution confocal images at 36% of embryogenesis show an early SOP and its initial lineage in the midline of the pedicellar domain following triple immunolabeling against epithelial cell-specific Lachesin (α-Lach, red), neuron-specific horseradish peroxidase (α-HRP, green), and the mitosis marker phosphohistone-3 (α-PH3, blue). Each label is shown in separate channels and then superimposed (merge). α-PH3 labels condensed chromatin in the large Lach-positive cell (white cross) confirming it is a mitotically active SOP. The SOP is associated with a cluster (clone) of four progeny, two of which (white stars) are HRP-positive and so represent differentiated neurons, while two (open/white arrowheads) are HRP-negative/Lach-positive at this stage. At 42% (**b**), a subset of three α-PH3-labeled SOPs (white crosses, outline dashed white) are visible in the pedicellar domain (red background), other mitotically active precursors (white dots) are located outside the pedicellar domain and are not associated with the JO. Two of the SOPs are associated with a smaller mitotically active precursor (outlined white) and HRP-positive neurons (white stars) from the clone project dendrites (open white arrowheads) apically. At 48% (**c**), α-PH3 labels a subset of six mitotically active precursors (white crosses, dashed white) within the Lach-domain (red background) as well as further precursors (white dots) outside the domain. Here three of the SOPs are accompanied by a smaller precursor (outlined white). HRP-positive dendritic processes (open white arrowheads) project apically, while bundled axons from pioneers and sensory cells of the flagellum project via ventral (vt) and dorsal (dt) tracts toward the antennal base. Lach-positive/HRP-positive co-labeled cell clusters (yellow) of developing pedicellar chordotonal organs (CO) are present. **d** Higher power confocal images show mitotically active SOPs (white crosses) at three successive ages in the developing JO. Each SOP generates a lineage (outlined dashed white) comprising three HRP-positive progeny (white stars) at 38%, four progeny at 40%, and 5 progeny at 42%. At 42%, the image orientation reveals a smaller proliferative cell (outlined white) associated with the lineage. Fasciculated dendrites (open white arrowheads) from the cluster project apically. **e** Confocal images show a ventral midline lineage from Johnston’s organ at three embryonic ages following labeling with neuron-specific HRP. Transverse optical sections show the lineage comprises two neuronal profiles (white stars) at 36%, three profiles at 38%, and five profiles at 41%. 3D reconstructions of the lineages (side views) show that progeny are consolidated into tightly bound cartridges. The scale bar in a represents 10 µm in a, d; 20 µm in b, e; 25 µm in c

At around 36% of embryogenesis (Fig. 3a), a single large Lach-positive/PH3-positive mitotically active cell is present in the midline region of the pedicellar domain where the initial clusters of the JO will form. This SOP is itself HRP-negative and at this age has generated a small lineage of four Lach-positive progeny, two of which are also HRP-positive and so represent the initial neurons of this cell cluster. At 42% (Fig. 3b), three PH3-positive mitotically active cells are generating lineages of the JO in the ventral Lach-positive domain of the pedicel. Toward mid-embryogenesis (48%, Fig. 3c), six PH3-positive progenitors are seen distributed throughout the ventral Lach-positive domain of the pedicel. The dendritic projections of their progeny project apically toward the border with the flagellum. Comparisons across a range of preparations of this age show that the number of active progenitors and lineages in the ventral Lach-positive pedicellar domain already matches the compliment of cell clusters belonging to the mature JO (see Fig. 7, c.f. Figure 1).

**Fig. 4:**
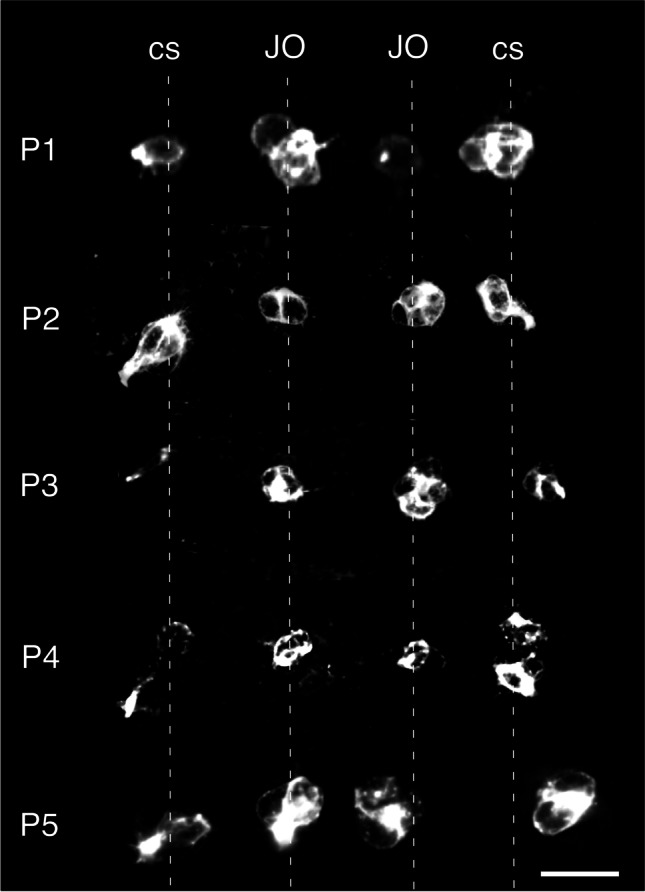
Confocal images from five repeat preparations (P1–P5) show initial neuronal lineages belonging to Johnston’s organ (JO) as well as campaniform sensilla (cs) in the ventral epithelium of the pedicel following α-HRP labeling at 39% of embryogenesis. Note the conserved locations of the cell clusters across preparations (vertical white dashed lines), consistent with there being an underlying topographic coordinate system to the epithelium. The scale bar represents 30 µm throughout

**Fig. 5:**
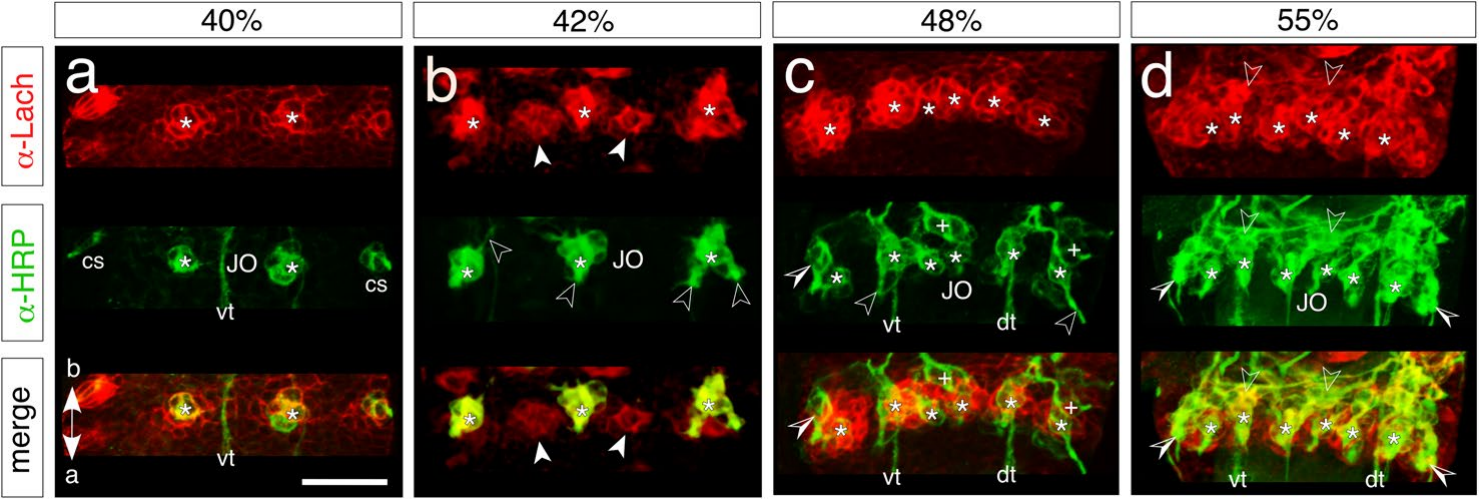
Development of the neuronal clusters Johnston’s organ (JO) in the pedicel at four successive ages following double-labeling against epithelial cell-specific Lachesin (red channels, top panels) and neuron-specific HRP (green channels, middle panels). Bottom panels show superposition of channels (merge). Confocal views are from ventral throughout. **a** At 40% of embryogenesis, two prominent clusters of Lach-positive cells (white stars) are present within the Lach domain (α-Lach). HRP-positive neurons (white stars, α-HRP) have differentiated and co-locate to each Lach cluster as superposition shows (merge, yellow). Neurons from campaniform sensilla (cs) have also differentiated. The ventral sensory tract (vt) from the flagellum transits the pedicel toward the antennal base. **b** At 42% of embryogenesis, five clusters of Lach-positive cells have formed in the Lach domain (α-Lach) of the pedicel. Superposition (merge, yellow) shows that three clusters of Lach-positive cells (white stars) co-locate to clusters of HRP-positive neurons (white stars, α-HRP), while two other clusters of Lach-positive cells (white arrowheads) do not yet contain differentiated neurons. Neurons have generated initial dendritic processes (open white arrowheads). **c** At 48% of embryogenesis, all five Lach-positive clusters in the Lach domain (α-Lach, white stars) now contain HRP-positive neurons (α-HRP, white stars) as superposition (merge, yellow) shows. Neurons have generated elongated dendritic processes (open white arrowheads), while campaniform sensilla (open/white arrowheads) and sensory cells of chordotonal organs (white crosses) have also differentiated. **d** At 55% of embryogenesis, six clusters of Lach-positive cells (α-Lach, white stars) are present in the pedicel. HRP-positive differentiated neurons of JO (α-HRP, white stars) co-locate to each of these clusters (merge, yellow). Neuronal clusters forming chordotonal organs (open white arrowheads) and campaniform sensilla (open/white arrowheads) are evident. The scale bar represents 60 µm in a, b; 55 µm in c; 65 µm in d

Along with the SOP, a smaller mitotically active cell can be seen in lineages at both 42 (Fig. 3b) and 48% (Fig. 3c) of embryogenesis. A time series imaged at higher resolution (Fig. 3d: 38%, 40%, and 42%) allows SOPs and their lineages to be analyzed more precisely. The data establishes that there is only a single large SOP associated with each progressively expanding lineage, which therefore constitutes a single clone. At each age, there is a smaller cell associated with the clone and in the same location with respect to the SOP. At two of these ages (38 and 42%), the cell in this location is mitotically active, while at 40%, it is between cell cycles. We can clearly not be sure that this smaller cell is the same cell each time, and it could equally represent a series of similar second-order precursors, each generated asymmetrically by the SOP before dividing.

3D confocal reconstructions following α-HRP labeling (Fig. 3e) document the age-dependent increase in neuronal numbers contributing to a lineage (36%:2 progeny, 38%:3 progeny, and 41%:5 progeny) and reveal that these neuronal progenies are consolidated into tightly bound cartridges.

### Topographic organization of the pedicellar epithelium

Our evidence up to this point is that a subset of Lach-positive/PH3-positive proliferative cells generates the neuronal progeny of the JO in the pedicellar domain. We reasoned that if the locations of these SOPs were fixed, then this should be reflected in a conserved distribution of their initial lineages in the epithelium, which in turn would argue for a topographic organization of the Lach-positive pedicellar domain.

Support for this hypothesis takes the form of a series of confocal images showing the initial cell clusters in the ventral epithelium of the pedicel in five repeat preparations of the same age (39%) after α-HRP labeling (Fig. 4). The data clearly show neuronal clusters of the JO as well as campaniform sensilla at remarkably conserved locations in the epithelium across these preparations. This is also the case for a range of other ages investigated (Suppl. Figure 1), from which we infer that the distribution of PH3-positive proliferative precursors generating these lineages is equally conserved and leads us to propose that the future cluster organization of the JO is based on a topographic organization of the epithelium.

### Developing neuronal clusters of Johnstons’organ

We investigated the developing pattern of cell clusters (clones) making up the JO in the pedicellar domain by labeling them with epithelial cell-specific α-Lachesin and neuron-specific α-HRP. At 40% of embryogenesis (Fig. 5a), two major clusters of Lach-positive cells are present in the ventral epithelial domain. Each cluster contains a lineage of differentiating neurons co-labeled by α-HRP, some of which are sprouting initial dendritic and axonal processes. By 42% of embryogenesis (Fig. 5b), five clusters of Lach-positive cells are present in the ventral epithelium, only three of which contain HRP-positive neurons at this stage. At 48% of embryogenesis (Fig. 5c), six Lach-positive cell clusters are now present, all of which contain HRP-positive neurons with significant axonal and dendritic processes. At 55% of embryogenesis (Fig. 5d), six clusters of cells are still present in the ventral Lach domain, all containing HRP-positive differentiated neurons. These neurons have generated extensive dendritic processes projecting apically toward the flagellum and axons projecting basally to the scape, where they fasciculate with the antennal nerve to the brain (see Fig. 1b).

The cluster number we see at each developmental stage reflects the number of active progenitor cells present, and by mid-embryogenesis matches that for the ventral subregion of the adult JO (c.f. Figure 1a). Subsequent development does not, therefore, appear to involve the generation of additional lineages.

**Fig. 6:**
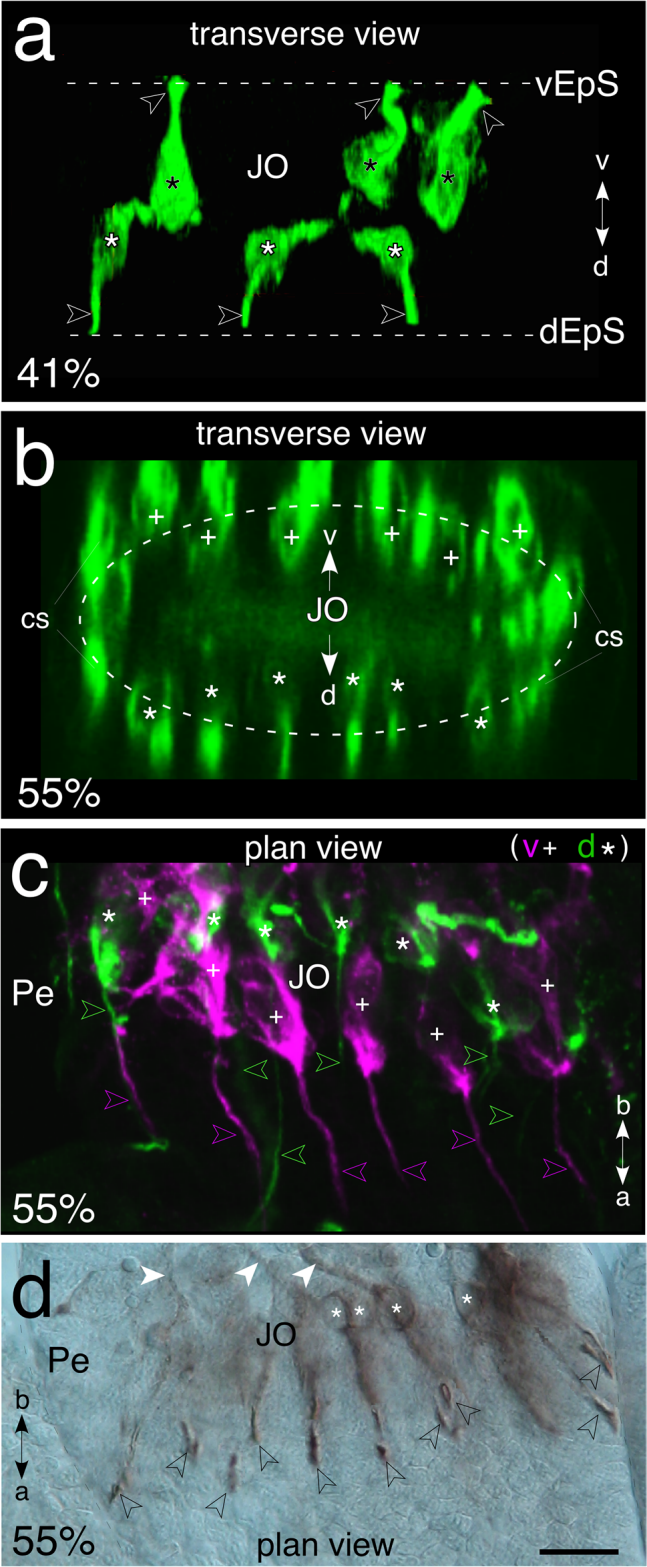
Cluster organization of Johnston’s organ during embryonic development following labeling with α-HRP. Antennal coordinates are a, apex; b, base; d, dorsal; v, ventral. **a** 3D reconstruction (transverse view) of the JO at 41% of embryogenesis reveals three ventral neuronal clusters (black stars) with dendrites (open white arrowheads) extending to the epithelium (vEp) and a mirror-symmetrical group of three dorsal cell clusters with dendrites (white/open arrowheads) extending to the dorsal epithelium (dEp). **b** 3D reconstruction (transverse view) of the JO at 55% of embryogenesis shows that there are now six ventral and six dorsal neuronal clusters organized circumferentially (dashed white oval) within the pedicel. Cell clusters of campaniform sensilla (cs) are present at the lateral edges of the pedicel (c.f. Gewecke 1972, 1979). **c** 3D confocal reconstruction of the JO at 55% of embryogenesis shows six ventral (white crosses, magenta) and six dorsal (white stars, green) cell clusters in the pedicel (Pe). Dendrites (magenta/green open arrowheads) from each cluster extend apically and in parallel for over 50 µm. **d** Image of the JO in the pedicel of a wholemount antenna viewed from ventral using Nomarski optics at 55% of embryogenesis following labeling with α-HRP and counterstaining with DAB. Clusters of HRP-positive neurons (white stars) direct axons (white arrowheads) basally toward the scape and scolopales apically to their insertion points (open black arrowheads) in the epithelium. The scale bar represents 25 µm in a, c; 20 µm in b; 30 µm in d

Our data above refer to events in the ventral epithelium, but there is a parallel, symmetrical development dorsally. In order to map the complete developmental pattern, we labeled cell clusters of the JO with α-HRP and then optically reconstructed these in 3D (Fig. 6). A transverse optical slice through the pedicel at 41% of embryogenesis shows three cell clusters with dendrites extending toward the ventral epithelial surface and a mirror-symmetrical group of three cell clusters with dendrites extending toward the dorsal epithelial surface (Fig. 6a). At 55% of embryogenesis (Fig. 6b), the number of ventral cell clusters has increased to six (c.f. Figure 5d), and a transverse view shows an equal number of dorsal clusters so that the complete JO is now organized circumferentially in the pedicellar epithelium. A 3D confocal reconstruction from a further preparation at 55% of embryogenesis (Fig. 6c) shows that scolopales from all six ventral and six dorsal cell clusters in the pedicel extend in parallel for over 50 µm toward the flagellum (c.f. Wolfrum 1990). We were able to visualize the scolopale insertion points in cap cells via Nomarski optics following α-HRP/PO labeling and DAB counterstaining (Fig. 6d).

**Fig. 7:**
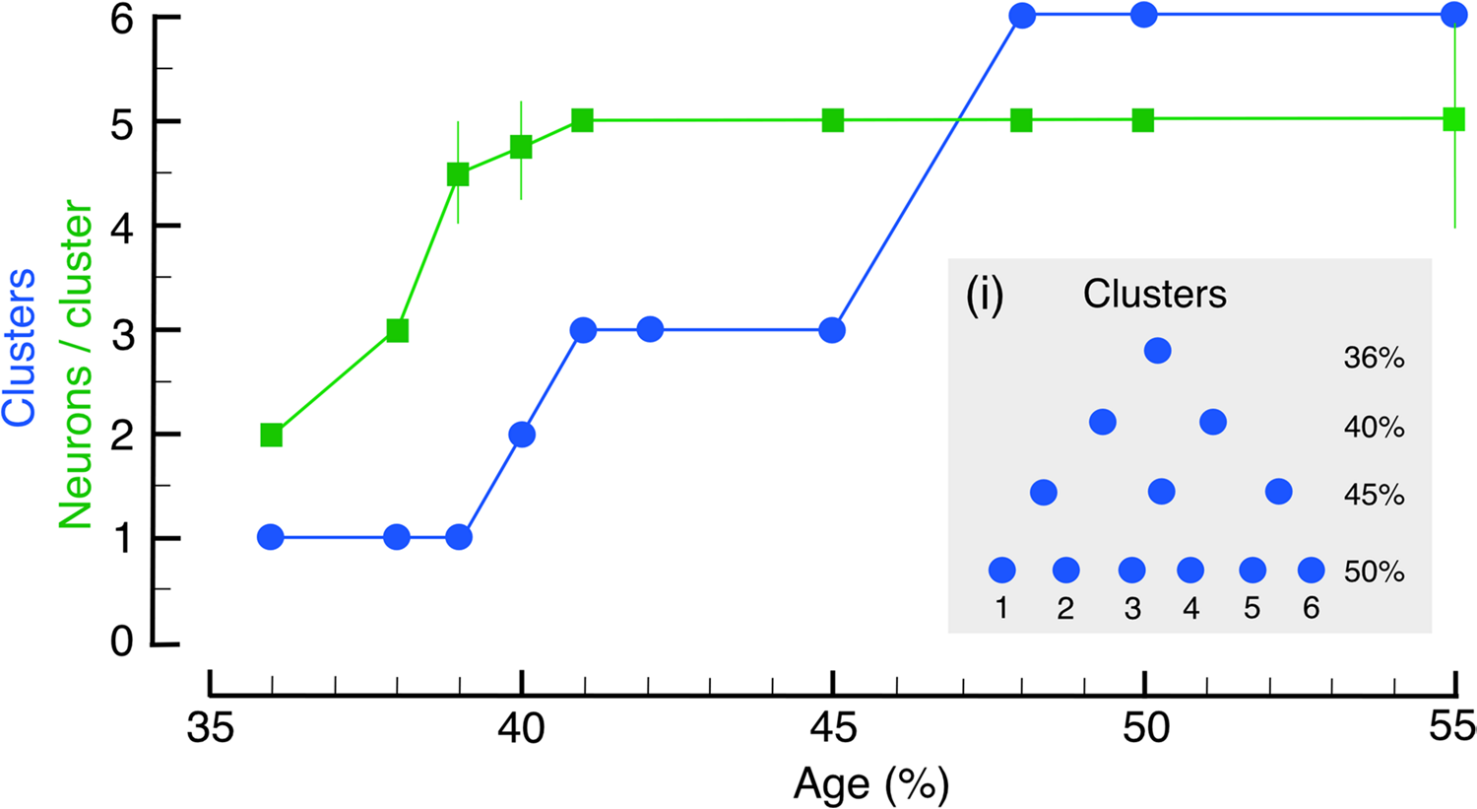
Graphical presentation of the number of HRP-positive clusters (blue) and the number of HRP-positive neurons/cluster (green) comprising Johnston’s organ at ages 35–55% of embryogenesis. As there was no significant difference in neuronal numbers between ventral and dorsal clusters at any given age, the data presented here derive from a representative cluster of the ventral midline. Data points for neurons/cluster (green) show means and standard deviations obtained from (*n*) preparations as follows: 36% (7), 38% (4), 39% (11), 40% (18), 41% (6), 45% (6), 48% (4), 50% (4), and 55% (4). Neuronal numbers increase in a saturating manner, stabilizing at 40%. Data points for numbers of clusters (blue) show means and standard deviations from the same preparations as above. Note that there was no variance whatever at any given age. Cluster numbers increase stochastically. The spatial distribution of clusters in the pedicel (inset **i**) suggests a binary increase with age and matches the pattern of mitotically active precursors and their lineages present in the Lach domain (c.f. Figures 3, 4, and 5)

The developing pattern of cell clusters, and the number of neurons contributing to each cluster of the JO up to mid-embryogenesis, are summarized graphically in Fig. 7. Our data reveal that for both ventral and dorsal pedicellar domains: (a) cluster numbers increase in a stepwise manner at each 5% of development; (b) cluster numbers are remarkably constant across all preparations, and between antennae, at each given age; (c) the spatial distribution of clusters within a domain changes in a binary fashion with age (Fig. 7, i).

The number of HRP-positive cells/clusters (Fig. 7) also increases with age, up to 41% of embryogenesis at which it saturates. The very small variance in neuronal number per cluster between preparations at a given age may be a function of our method. We staged preparations to the nearest 1% (5 h) of developmental time so that some embryos could have been slightly advanced or retarded temporally compared to others. There was also no significant difference in neuronal numbers comprising the ventral and dorsal cell clusters for a given age (data not shown). It should be emphasized that our data for cell numbers here are based exclusively on HRP-positive, differentiated, and neurons. Double-immunolabeling against neuron-specific HRP and the sensory cell marker Lazarillo, however, reveals the presence of one or more extrinsic cells associated with each cluster (Suppl. Figure 2). These extrinsic cells are exclusively HRP-negative/Laz-positive over the age spectrum we tested, and their identity must be established in a later study.

## Discussion

Johnston’s organ is a prominent sensory structure of the antenna across a wide spectrum of insect species and has been universally found to function in audition (Toh 1981; Toh and Yokohari 1985; Jeram and Pabst 1996; Göpfert and Robert 2001a, b; 2002; Göpfert et al. 2002; Todi et al. 2004; Boekhoff-Falk 2005; Eberl and Boekhoff-Falk 2007; Jarman 2014). In the locust *Schistocerca gregaria*, the JO is located in the pedicellar segment of the antenna (Gewecke 1972, 1979) and, as with other sensory structures in hemimetabolous insects (see Anderson 1973; Chapman 1982), must develop during embryogenesis in order to be functional on hatching. We show here that the cellular organization of the JO at mid-embryogenesis already strongly resembles that of the adult (Figs. 1, 5, and 6) and is consistent with the timeframe over which the sensory complement of the tympanal ear forms in orthopteroid insects (Meier and Reichert 1990; Klose 1991). It is likely that maturation of the JO will take place during late embryonic and postembryonic development and involve changes in mechano-sensitivity as reported for the tympanal ear of orthopteroid insects (Ball and Young 1974; Ball and Hill 1978; Ball 1979; Michel and Petersen 1982). Mechanosensitivity of the antennal chordotonal sense organ (CHO) in *Drosophila* is regulated by a spectrum of transcriptional and motor proteins (see Göpfert and Robert 2001a; Boekhoff-Falk 2005; Todi et al. 2005; Eberl and Boekhoff-Falk 2007), including *atonal*, which is required for the formation of the joint associated with the CHO and where the loss of *ato* function renders the antennal receiver insensitive to sound (Göpfert et al. 2002). It is speculative but plausible that these mechanisms are conserved across insect species.

### Topographic organization of the pedicellar epithelial domain

The scape, pedicel, and flagellum of the locust antenna represent true segments, and their development is regulated by molecular mechanisms homologous to those forming other head and body appendages (see Gibson and Gehring 1988; Casares and Mann 1998). Accordingly, antibody labeling shows that common patterns of cell surface epitopes such as Annulin (Bastiani et al. 1992; Boyan et al. 2018) and Lachesin (Karlstrom et al. 1993; Boyan and Ehrhardt 2020) are present in their epithelia. Lachesin expression in the embryonic pedicel takes the form of a single stripe (Fig. 2) within which putative sense-organ precursors (SOPs) generating the cell clusters of the JO are located (Figs. 2d and 3). The initial neuronal progeny of these SOPs are seen to occupy remarkably conserved locations within the pedicellar domain (Fig. 4; Suppl. Figure 1), consistent with a predetermined position and suggesting a topographic organization of the Lach-positive epithelial domain of the pedicel. Our data, therefore, correlate with the position-specific coordinate system for neuroectodermal progenitors of the central nervous system of the locust and *Drosophila* (Doe et al. 1991; Doe 1992), as well as for sensory epithelia such as the retina of *Drosophila* (see Fischbach and Hiesinger 2008). Whether the position of progenitors in the pedicel of the locust antenna is regulated by mechanisms homologous to those identified in *Drosophila* (*Seven up*, *Prospero*, *gooseberry-distal*: see Skeath et al. 1995) awaits clarification.

### Neuronal lineages

As the sense-organ precursors (SOPs) generate their lineages, their conserved locations translate into the subsequent organization of cell clusters making up the JO (Figs. 3, 4, and 6). We found the number of clusters contributing to the JO at any given age to be remarkably constant, with no variance at all across 18 preparations at 40% of embryogenesis, for example (Fig. 7). We detected only one large SOP associated with each lineage, indicating that the progeny represent a single clone, as in the cockroach (Blöchl and Selzer 1988) and *Drosophila* (Campos-Ortega and Hofbauer 1977; Lawrence and Green 1979). In *Drosophila*, the selection and specification of SOPs are regulated by a range of factors, including *homothorax*, *cut*, *atonal,* and Delta-Notch signaling (see Artavanis-Tsakonas and Simpson 1991; Jarman et al. 1993, 1995; Jarman 2014 for details), factors which have been shown to be conserved (see Singhania and Grueber 2014) and so may also play a role in determining SOP identity in other species. Each clone would then derive from successive asymmetric cell cycles of an SOP, as has been proposed for antennal pioneers in the locust (Boyan and Ehrhardt 2017), Johnston’s organ in *Drosophila* (Jarman 2014), and visual neurons of the medulla in *Drosophila* (Apitz and Salecker 2014). In order to determine if the neuronal complement of a clone in the locust is traceable to the smaller precursor accompanying each SOP (Fig. 3b–d), which would then constitute part of the pIIb/pIIIb path as in the homologous structure in *Drosophila* (Jarman 2014), we need to be able to identify such precursors individually (c.f. Pearson and Doe 2003). Application of pulsed labeling with Edu/BrDU, along with intracellular dye injection, both methods which have previously allowed the ontogeny of pioneer neurons at the antennal tip of the embryonic locust to be established (Boyan and Ehrhardt 2017), may help resolve lineage questions in cell clusters of the JO.

Taken together, our data are consistent with the JO in the locust possessing a type I sensory organization in common with other internal chordotonal organs for stretch or vibration (see Yack 2004; Singhania and Grueber 2014). Indeed, comparative data reveal a remarkably similar circumferential organization of cell clusters in both the JO of the embryonic locust (Fig. 6b) and the phylogenetically distant adult ant (Grob et al. 2021, Fig. 4), consistent with a conserved structural requirement for detecting and encoding these environmental signals.

### Cartridge composition

Our confocal reconstructions show that the neurons of each lineage of the JO are organized into cartridges (Figs. 3e and 6; Suppl. Figure 2), whose organization is reminiscent of that of retinal photoreceptors in the fly (see Strausfeld 1976; Meinertzhagen and Hanson 1993; Jarman et al. 1995). Indeed, the fact that in *Drosophila atonal* functions in the initial development of Johnston’s organ, stretch receptors, and the eye, has led to the suggestion that these organs derive from an ancestral *atonal*-dependent protosensory organ (Niwa et al. 2004).

The final number of HRP-positive cells contributing to a cartridge of the JO in the locust is five (Fig. 7), as it is in the adult scolopendrium of the connective CHO in the pedicel of the cockroach (Blöchl and Selzer 1988), the mosquito (Schmidt 1967), or olfactory sensilla on moth antennae (Keil and Steiner 1990; Keil 1997). Consistent with this, comparative data suggest the SOP generates the clonal cartridge comprising cap cell, attachment cell, scolopale cell, ligament cell, and neurons according to a ground plan for sensillum development which is conserved across species (see Lai and Orgogozo 2004; Jarman 2014). Given this common organization of sensory cartridges across species, the mechanisms for determining cell fates (see Fischbach and Hiesinger 2008) and assembling the postsynaptic cell complement in sensory cartridges (see Huang et al. 1998) reported for *Drosophila* may also be conserved but have not yet been demonstrated in the locust.

When we examined the sensory clusters of the JO in the locust via double-labeling against Lazarillo, a GPI-linked cell surface lipocalin expressed by sensory cells in both the locust and *Drosophila* (Ganfornina et al. 1995; Sánchez et al. 1995), and neuron-specific HRP (Jan and Jan 1982), we found additional Lazarillo-positive/HRP-negative cells associated with each cartridge (Suppl. Figure 2). Such non-neuronal cells could represent the attachment or ligament cells found in the JO of *Drosophila* (Jarman 2014) or glia-like accessory cells common to sensory units associated with mechano- and olfactory sensilla of insect antennae (Blöchl and Selzer 1988; Keil and Steiner 1990; Keil 1997). Their identity in the locust is still unclear as previous experiments with the glia-specific homeobox gene repo failed to find an expression compatible with the developing cell clusters of the JO (Boyan and Williams 2004, 2007). Additional labeling, for example with α-Tubulin, which in the cockroach specifically stains the attachment cell, or fluorescent phalloidin, which exclusively stains the scolopale (see Wolfrum 1990), may provide insights into their identity, as would a future ultrastructural analysis.

## Supplementary Information

Supplementary Fig. 1 Confocal images show neuronal lineages belonging to Johnston’s organ (JO) as well as campaniform sensilla (cs) and chordotonal organs (white arrowheads) in the ventral epithelium of the pedicel following α-HRP labeling in three repeat preparations (P1–P3) at each of 36%, 38%, 41% of embryogenesis. Note the conserved locations of the cell clusters across preparations (vertical white dashed lines; c.f. Fig. 4) consistent with there being an underlying topographic coordinate system to the epithelium. Scale bar represents 50μm throughout (PNG 378 kb)

High resolution image (TIF 1265 kb)

Supplementary Fig. 2 Extrinsic cells associated with sensory clusters of Johnston’s organ (JO). Confocal images of a sensory cluster in transverse view from two preparations (**a**–**c**, **d**–**f**) both at 40% of embryogenesis following double-labeling against neuron-specific HRP (**a, d;** α-HRP, green) and sensory-cell specific Lazarillo (**b, e;** α-Laz, red). In each case the cluster has a cartridge-like form and comprises four neuronal profiles (white stars) which are co-labeled by α-HRP and α-Laz (**c, f**; merge, yellow) and so represent differentiated sensory neurons. Associated with the cluster are the profiles of further cells (open white arrowheads) which are HRP-negative/Laz-positive. The identities of such non-neuronal cells remain to be determined. **g.** 3D reconstruction of a cell cluster at 40% in side view following double-labeling against α-HRP (green) and α-Laz (red) and subsequent superposition of channels (merge, yellow). An HRP-negative/Laz-positive extrinsic cell (red, open white arrowhead) is associated with four HRP-positive/Laz-positive sensory neurons (yellow, white stars). Coordinates point to the dorsal and ventral epithelial surfaces. Scale bar represents 25μm in a-f; 8μm in g (PNG 509 kb)

High resolution image (TIF 5112 kb)

## Data Availability

Core data supporting this study are archived with Dr. E.E. Ehrhardt, AG Ito, Institute of Zoology, Universität zu Köln, Zülpicher Str. 47b, 50,674 Cologne, Germany and can be viewed on request.
