# Supplementary figures and images for: Early embryonic development of Johnston’s organ in the antenna of the desert locust Schistocerca gregaria
Source: Dev Genes Evol. 2022 Sep 23;232(5-6):103–13. doi: 10.1007/s00427-022-00695-2 (PMC9691482; doi:10.1007/s00427-022-00695-2)

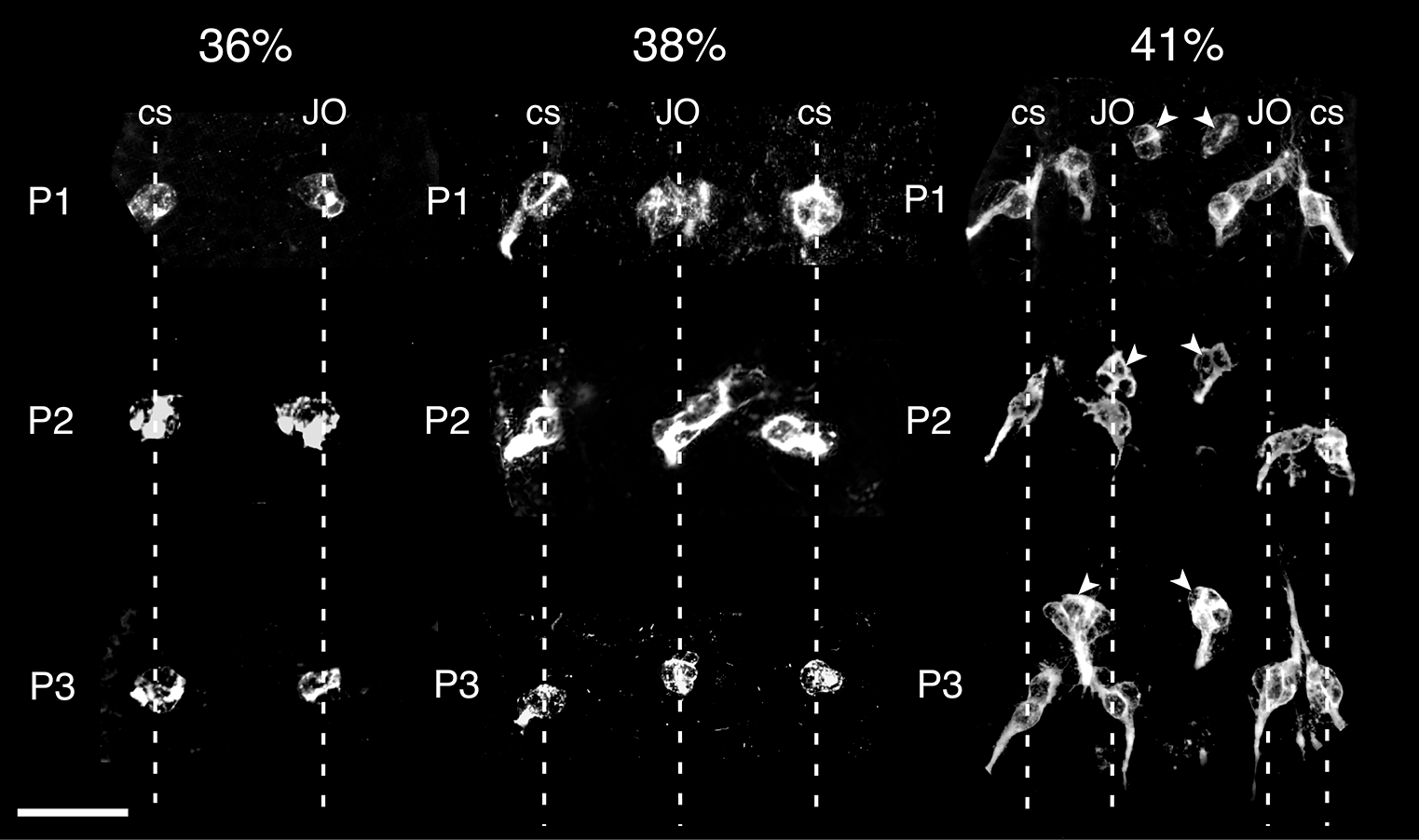

Supplement: Supplementary file 1 — Confocal images show neuronal lineages belonging to Johnston’s organ (JO) as well as campaniform sensilla (cs) and chordotonal organs (white arrowheads) in the ventral epithelium of the pedicel following α-HRP labeling in three repeat preparations (P1–P3) at each of 36%, 38%, 41% of embryogenesis. Note the conserved locations of the cell clusters across preparations (vertical white dashed lines; c.f. Fig. 4) consistent with there being an underlying topographic coordinate system to the epithelium. Scale bar represents 50μm throughout (PNG 378 kb) [file 427_2022_695_Fig8_ESM.png]

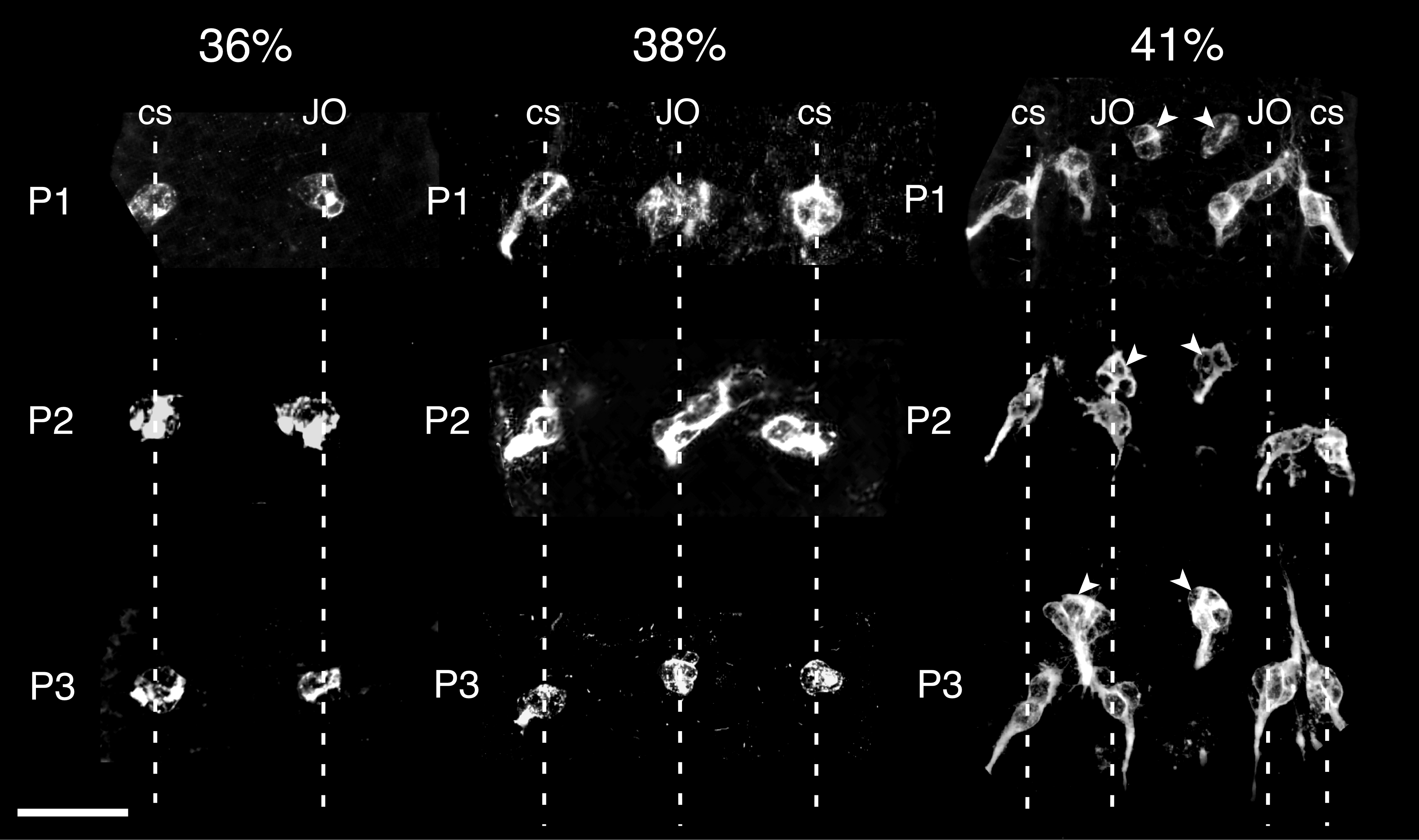

Supplement: Supplementary file 2 — High resolution image (TIF 1265 kb) [file 427_2022_695_MOESM2_ESM.tif]

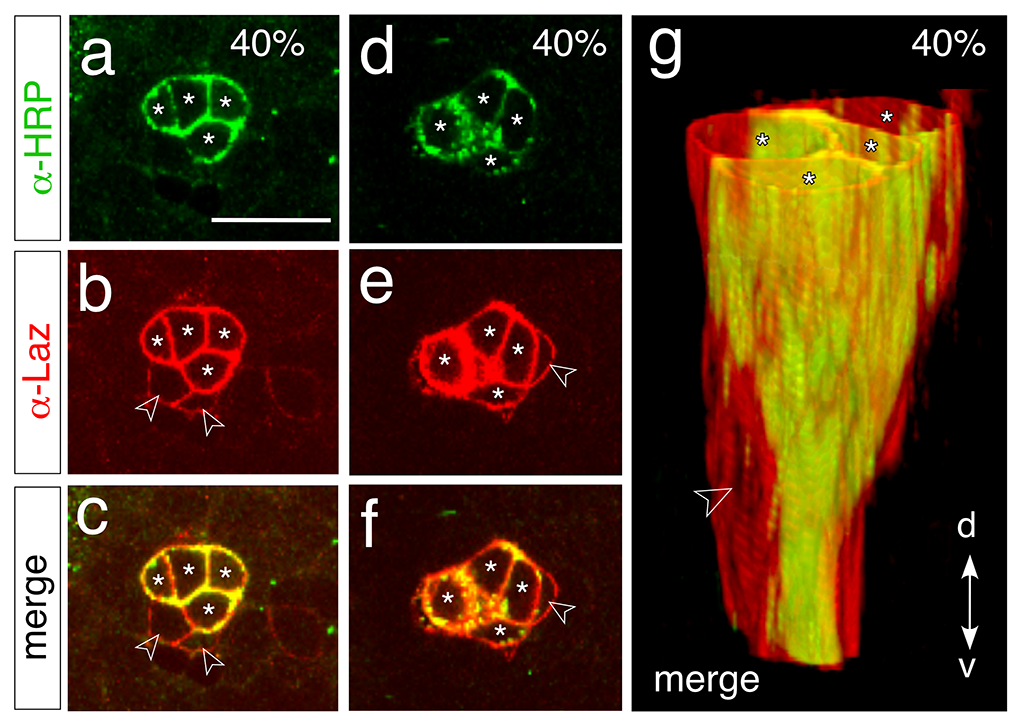

Supplement: Supplementary file 3 — Extrinsic cells associated with sensory clusters of Johnston’s organ (JO). Confocal images of a sensory cluster in transverse view from two preparations (a–c, d–f) both at 40% of embryogenesis following double-labeling against neuron-specific HRP (a, d; α-HRP, green) and sensory-cell specific Lazarillo (b, e; α-Laz, red). In each case the cluster has a cartridge-like form and comprises four neuronal profiles (white stars) which are co-labeled by α-HRP and α-Laz (c, f; merge, yellow) and so represent differentiated sensory neurons. Associated with the cluster are the profiles of further cells (open white arrowheads) which are HRP-negative/Laz-positive. The identities of such non-neuronal cells remain to be determined. g. 3D reconstruction of a cell cluster at 40% in side view following double-labeling against α-HRP (green) and α-Laz (red) and subsequent superposition of channels (merge, yellow). An HRP-negative/Laz-positive extrinsic cell (red, open white arrowhead) is associated with four HRP-positive/Laz-positive sensory neurons (yellow, white stars). Coordinates point to the dorsal and ventral epithelial surfaces. Scale bar represents 25μm in a-f; 8μm in g (PNG 509 kb) [file 427_2022_695_Fig9_ESM.png]

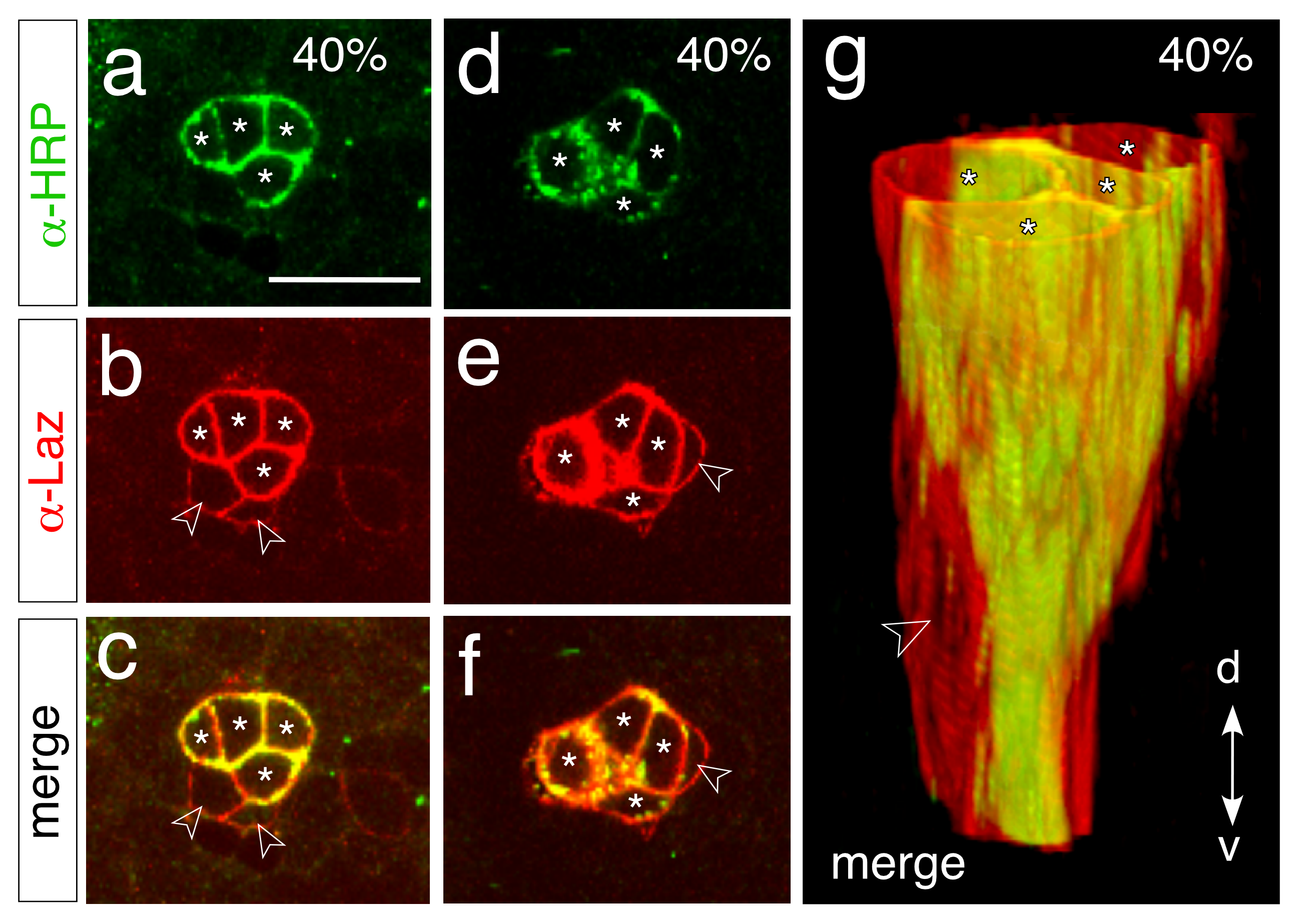

Supplement: Supplementary file 4 — High resolution image (TIF 5112 kb) [file 427_2022_695_MOESM4_ESM.tif]
